# A Review and Hypothesized Model of the Mechanisms That Underpin the Relationship Between Inflammation and Cognition in the Elderly

**DOI:** 10.3389/fnagi.2019.00056

**Published:** 2019-03-13

**Authors:** Masoumeh Tangestani Fard, Con Stough

**Affiliations:** Centre for Human Psychopharmacology, Swinburne University of Technology, Melbourne, VIC, Australia

**Keywords:** cognitive aging, cognitive decline, inflammation, inflamm-aging, vascular inflammation, neuroinflammation

## Abstract

Age is associated with increased risk for several disorders including dementias, cardiovascular disease, atherosclerosis, obesity, and diabetes. Age is also associated with cognitive decline particularly in cognitive domains associated with memory and processing speed. With increasing life expectancies in many countries, the number of people experiencing age-associated cognitive impairment is increasing and therefore from both economic and social terms the amelioration or slowing of cognitive aging is an important target for future research. However, the biological causes of age associated cognitive decline are not yet, well understood. In the current review, we outline the role of inflammation in cognitive aging and describe the role of several inflammatory processes, including inflamm-aging, vascular inflammation, and neuroinflammation which have both direct effect on brain function and indirect effects on brain function *via* changes in cardiovascular function.

## Introduction

**Figure 1:**
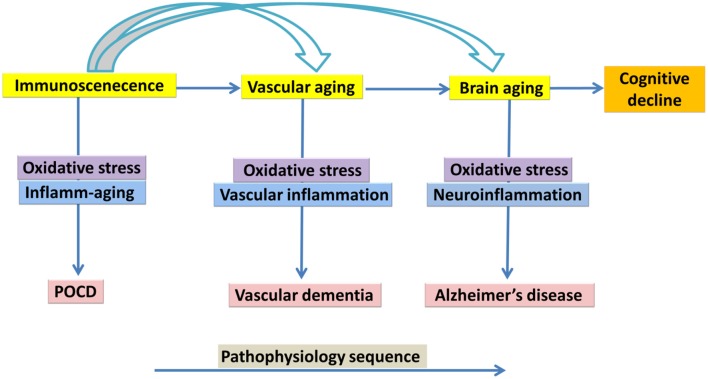
Immunosenescence, vascular aging, brain aging in association with cognitive decline, a suggested model of underlying mechanism.

Life expectancies have increased considerably since the 1950s in developed countries. For example, the population of octogenarians in developed countries has increased four-fold, the population of nonagenarians eight-fold, and the population of centenarians 20-fold. Aging is a progressive decline in the physiological integrity of different organs of the human body, which leads to impaired body function and enhanced vulnerability to death. This deterioration is a crucial risk factor for the main human pathologies, including neurodegenerative diseases, cardiovascular disorders, cancer and diabetes (López-Otín et al., 2013). To date, several variations of innate and acquired immunity have been observed in the elderly. These alterations have been generally, explained as a deterioration of immunity, which has been referred to as immunosenescence and is characterized *via* chronic inflammatory conditions. Immunosenescence refers to increased susceptibility of the elderly to infection and may be explained in terms of molecular and cellular mechanisms responsible for inflammatory age-associated disorders (Larbi et al., 2008; Caruso et al., 2009). A better understanding of immunosenescence and the development of novel strategies to counteract it are necessary, not only for anti-aging strategies aimed at preventing or slowing down cognitive aging but, more notably with the aim of prolonging healthy life, through preventing infectious and age-associated disorders and improving the quality of life in later years (Candore et al., 2008; Jirillo et al., 2008; Larbi et al., 2008; Caruso et al., 2009; Holmes et al., 2009; Trollor et al., 2010; Barrientos et al., 2015; Di Benedetto et al., 2017). Cognitive aging is characterized by a decline in memory and other cognitive processes, changes in behaviors and impaired ability to live an independent and high functioning life (Cunningham and Hennessy, 2015). In the current review article, we bring together different biological processes related to inflammation within the context of cognitive aging. There have been few theoretical models of the molecular and cellular mechanisms of cognitive decline, with most of the literature focusing on abnormal aging and cognitive disorders of aging such as Alzheimer’s Dementia (AD; Changeux and Dehaene, 1989; Miller and Cohen, 2001; Zlokovic, 2005; Bishop et al., 2010). The cytokine model of cognitive function explained by McAfoose and Baune (2009) emphasized the important role of cytokines in cognitive process at the molecular level such as in synaptic plasticity, neurogenesis, and neuromodulation, which may subserve learning, memory, and other cognitive processes. This cytokine-mediated model of cognitive processes has been proposed to be causative in terms of longer-term pathogenesis related to some neuropsychiatric disorders such as AD and Major Depression (McAfoose and Baune, 2009) but there is a lack of clarity in terms of how some of these processes may affect cognitive aging. In this review, we outline the involvement of three main aging features of the central nervous system (CNS) that underpin cognitive decline (Figure 1). Specifically, we present a model of cognitive aging that comprises three main aging features of the CNS, including immunosenescence, vascular aging, and brain aging and we briefly review the role of each of these components in terms of changes in cognition with increasing age.

## Cognition

Cognition refers to mental processes that are often measured in terms of our ability to allocate attention, recall information, to perceive relationships as well as the ability to think locally and abstractly amongst other cognitive domains. Some of these cognitive domains decrease, as we get older (Christensen, 2001; Singh-Manoux et al., 2012). In particular, memory and processing speed appear to be more sensitive to age than other cognitive domains (Salthouse, 1996; Christensen, 2001). A reduction in cognitive function affects more than 50% of people over 60 years of age (Skaper et al., 2014). Dementia is a generic term that encompasses several diseases with different pathologies such as AD, vascular dementia (VD), frontotemporal dementia, and dementia with Lewy bodies. Their common characteristic is a progressive reduction in cognitive performance, which leads to functional dependency and death (Gao et al., 2016). However, it is unclear which biological processes underpin these changes. Some researchers have proposed a linkage between inflammatory processes and cognition. Although most of this research has been derived from animal studies, the results of which could also be applied to understanding human conditions such as cognitive aging. These investigations have emphasized a close association between some aspects of the immune system, processes at the level of the neuron and vascular systems (Zlokovic, 2005; McAfoose and Baune, 2009; Grammas, 2011; Broussard et al., 2012; Davenport et al., 2012; Kousik et al., 2012; Barrientos et al., 2015; Di Benedetto et al., 2017; Tarantini et al., 2017). Interestingly, a recent review by Gauthier et al. (2018) argued for the importance of considering the interaction of several factors involved in age-associated cognitive decline (particularly AD) such as vascular small vessel disease, neuroinflammation and Lewy body pathology (Gauthier et al., 2018). Vijayan and Reddy (2016) also argued that stroke was a major risk factor contributing to AD and VD through several cellular and molecular changes including inflammation, oxidative stress, mitochondrial dysfunction, vascular changes and marked changes in brain proteins.

## Immunosenescence

The remarkable development of human survival and lifespan to well beyond childbearing ages has been completely “unpredicted” *via* evolution (Baylis et al., 2013). As a result, the human immune system is exposed to significant additional antigenic exposure outside the forces of natural selection (De Martinis et al., 2005; Franceschi, 2007; Franceschi et al., 2007). The immune system is highly effective for the first 40 years of life and after that, similar to all other organs and systems of the body undergoes a process of senescence and certain features begin to reveal effectual reduction (Piazza et al., 2010; Salvioli et al., 2013). The immunity begins to exhibit negative effects on human aging (antagonistic pleiotropy) leading to gradual systemic failures (De Martinis et al., 2005; Franceschi, 2007; Franceschi et al., 2007). The process of senescence causes a progressive reshaping of its functions in a pervasive process, which influences almost all the compartments of the immune system, particularly the branch in control of the acquired immunity (Salvioli et al., 2013). The immune system during aging declines in efficiency and reliability resulting in greater susceptibility to pathological conditions as a consequence of chronic inflammatory responses, for instance, Alzheimer’s disease, cardiovascular disease, auto-reactivity as well as an enhanced vulnerability to infectious disease (Baylis et al., 2013). These variations are further compounded by a reduction in responsiveness-impaired communication among all cells of the immune system. The overall alteration of the immune system during aging is termed “immunosenescence” and has a multifactorial etiology (Weiskopf et al., 2009). Immunosenescence of the acquired immune system includes the involvement of the thymus and reduced responsiveness to new antigen load, due to reduced naïve: memory cell ratio and expansion of mature cell clones (Baylis et al., 2013; Müller and Pawelec, 2015). Thymic output decreases with age resulting in reduced T-cell repertoire and enhanced oligoclonal expansion of memory and effector-memory cells (Pawelec, 2012). This imbalance leads to a reduced ability to clear novel pathogens (prolonging infection duration) as well as an elevation in functionally distinct T-cell populations that have an amplified pro-inflammatory phenotype (Weiskopf et al., 2009). Immunosenescence of the innate immune system is mainly defined by the reduction in cellular superoxide production and capability for phagocytosis (Pawelec, 2012). Of the innate immune system, monocytes and macrophages are assumed to lead to inflamm-aging (cellular exhaustion) more than any other cell type. Monocyte variations with age can cause inflamm-aging *via* reduced function and a functional shift against a proinflammatory phenotype (Shaw et al., 2010).

## What Is Inflamm-Aging?

Another striking characteristic feature of immunosenescence is an increase in cellular production of proinflammatory mediators, such as tumor necrosis factor-α (TNF-α), interleukin-6 (IL)-6 and IL-1β in serum’s individuals (Zanni et al., 2003; Salvioli et al., 2013) and has been indicated as inflamm-aging the chronic sub-clinical elevated production of pro-inflammatory mediators typical of elderly (Blagosklonny and Hall, 2009; Blagosklonny, 2010). Inflamm-aging is a consequence of a cumulative lifetime exposure to antigenic load due to both clinical and sub-clinical infections as well as non-infective antigens (Baylis et al., 2013). It is believed to be related to several age-associated disorders sharing a similar inflammatory basis. However, recent research indicates that inflamm-aging is at least in part independent from immunological stimuli. In addition, centenarians who prevented or delayed major inflammatory disorders display elevation in inflammatory mediators (Salvioli et al., 2013). The result of the inflammatory response is tissue damage and the release of reactive oxygen species (ROS), which can lead to oxidative damage and in turn stimulate the production of increased levels of cytokines, principally from cells of the innate immune system (Cannizzo et al., 2011) as well as the acquired immune system. These events initiate a vicious cycle in which the immune system is remodeled favoring a chronic pro-inflammatory response where, healing responses, pathophysiological variations, and tissue damage occur at the same time. Irreversible molecular and cellular damage, which is not clinically noticeable, gradually accumulates over decades (Baylis et al., 2013). Theoretically, the process of inflamm-aging could account for the increased frequency of inflammation-based pathologies, which occur with increased age (e.g., neurodegeneration, cardiovascular diseases, arthritis, type II diabetes, and several types of cancers). The hypothesis that aging is driven *via* unnecessary inflammatory responses stems from the concept of inflamm-aging and is consistent with the recent theory of aging as a quasi-program (Blagosklonny and Hall, 2009; Blagosklonny, 2010).

Another cellular phenomena, relevant to immunosenescence and inflammation is “cellular senescence” which is a hallmark of aging and has been reported in a large number of studies in which the mechanisms have been extensively reviewed (Campisi and d’Adda di Fagagna, 2007; Collado et al., 2007; Collado and Serrano, 2010; Kuilman et al., 2010; Salama et al., 2014; van Deursen, 2014; Walters et al., 2016). Cellular senescence can occur in almost all cell types that are capable of cell division (Bitto et al., 2010; Coppé et al., 2010b; Chinta et al., 2015). Recent evidence in epithelial cells and fibroblasts have revealed that cellular senescence is mediated through a large increase in the production of 40–80 factors that play a vital role in intercellular signaling (Coppé et al., 2008, 2010a; Young and Narita, 2009; Chinta et al., 2015). The release of these series of factors has been termed “senescence-associated secretory phenotype,” or SASP and they generally increase at mRNA levels (Coppé et al., 2008) and enhance a wide array of proteases, chemokines, growth factors, and cytokines. SASP proteins that are characterized as inflammatory stimulators, include IL-8, IL-6, IL-1, granulocyte macrophage colony stimulating factor, monocyte chemotactic protein-2 (MCP-2), MCP-3, matrix metalloproteinase-1 (MMP-1), MMP-3, growth regulated oncogene-α, and several Insulin-like growth factor-binding proteins (Kumar et al., 1992; Wang et al., 1996; Coppé et al., 2008) which are among the most largely secreted SASP factors and potentially can cause or aggravate, age-related pathology, both degenerative and hyperplastic (Chinta et al., 2015). Therefore, senescent cells are a source of chronic inflammation senescence/inflamm-aging during the aging process (Freund et al., 2010). Some other studies also indicated that inflammation could exacerbate the biological aging and cellular senescence, which may be a cause for the loss of cognitive function (Panossian et al., 2003; Honig et al., 2006; Ma et al., 2013).

## Inflamm-Aging, Cellular Senescence and Cognitive Aging

The relationship between inflamm-aging and cognitive aging has been reported in several human studies (Dik et al., 2005; de Rooij et al., 2007; Schram et al., 2007; Marioni et al., 2009; Kim et al., 2015). For instance, Trollor et al. (2010) argued for a relationship between inflamm-aging and mild cognitive impairment (MCI), in the Sydney memory and aging study cohort, a longitudinal study of 1,037 Australians aged 70–90 years. The findings showed that the concentration of TNF-α and serum amyloid A, were higher in individuals with MCI compared with cognitively normal participants (Trollor et al., 2010). Three hundred community-dwelling individuals with mild to severe Alzheimer’s disease were tested on cognitive measures and inflamm-aging. An increase in the serum level of TNF-α and a two-fold increase in the rate of cognitive decline over 6 months were observed in around half of all study participants. In the baseline group, high levels of TNF-α was associated with a four-fold enhancement in the rate of cognitive decline. Individuals with low serum levels of TNF-α showed no cognitive decline over the 6 months (Holmes et al., 2009). In another study by Yaffe et al. (2003), a group of 3,031 White and African-Americans with mean age of 74 years were assessed in terms of cognitive function and inflamm-aging. Individuals in the highest tertile for C-reactive protein (CRP) or IL-6 had nearly two points lower scores on the Modified Mini-Mental State Examination (3MS) at baseline. These scores then declined further over the 2 years of the study in comparison with those with the lowest tertile for CRP or IL-6. Participants with the highest inflammatory mediators tertile were also more likely to have cognitive decline compared with participants with the lowest tertile for IL-6 and for CRP but not for TNF-α (Yaffe et al., 2003).

**Figure 2:**
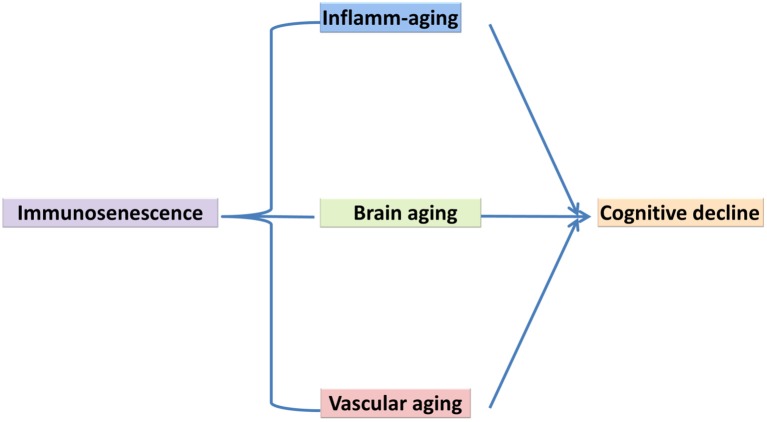
Immunosenescence is a key mechanism underlying cognitive decline.

While research on the cellular senescence in brain aging is at early stages, the role of cellular senescence in peripheral tissues during several age-related pathologies has been more frequently explored (Chinta et al., 2015). Animal studies have been published on brain cellular senescence and cognitive aging (Cho et al., 2015; Tarantini et al., 2017), for example a study by Li et al. (2018) reported on D-galactose-induced aging in mouse model and showed that Zicao (Acetyl shikonin) treatment significantly reduced hippocampus senescence and cognitive impairments through upregulating the expression of SIRT1 and suppressing inflammatory cytokines such as IL-1β and TNF-α (Li et al., 2018). Ungvari et al. (2017) reported that whole brain irradiation-induced accelerated brain senescence is notably associated with cerebromicrovascular dysfunction and cognitive decline. Parisotto et al. (2016) showed that Melatonin treatment reduced cellular senescence and oxidative damage in the hippocampus of a mouse model of down syndrome. Studies on human brain samples have also indicated an increase in DNA-protein kinase catalytic subunit and incorporating phosphorylated histone, γH2AX (which exhibited a neuronal DNA damage response) is correlated with cognitive impairment (Simpson et al., 2015) (Figure 2).

## Immunosenescence and Oxidative Stress

The majority of age-related disorders are associated with a low level of chronic inflammation (De la Fuente and Miquel, 2009; Cannizzo et al., 2011). Currently, it is not yet, fully understood whether inflammatory responses lead to the development of degenerative chronic disorders or whether the chronic pathologies can lead to inflammatory response observed during aging. Regardless of the cause-effect relationship among age-dependent disorders and inflammation, oxidative stress is known to play a main role in maintaining the chronic inflammation or inflamm-aging observed in aging and age-dependent disorders (De la Fuente and Miquel, 2009). Toll-like-receptors and the Nalp-3 inflammasome are two main molecular pathways which develop inflammatory responses through oxidative damage produced by free radicals (Cannizzo et al., 2011). Moreover, increases in oxidative stress can lead to telomere length attrition and cellular senescence which may be associated with cognitive decline (Ma et al., 2013).

The idea of ROS involvement in aging process, dates back to 1995 when Harman suggested the “Free Radical Theory of Aging” or “oxidative stress theory of aging,” referring to accumulation of free radicals during aging process which could cause biomolecule damage and the development of pathological condition (Harman, 1992, 2006; Harvey et al., 2015; Black et al., 2017). Free radicals are molecules with unpaired electrons which are mainly unstable (Cannizzo et al., 2011) and increase the non-enzymatic oxidation of biomolecules (proteins, carbohydrates, lipids and nucleic acids; Halliwell, 2009; Buonocore et al., 2010; Hamanaka and Chandel, 2010). Two main biochemical mechanisms link immunosenescence to oxidative stress: (i) a decline in cellular functions because of oxidative damage in protein, lipid and carbohydrate; and (ii) cellular apoptosis followed by accumulation of oxidized molecular aggregates. The elevation in free radicals volume in several aging cells has also been observed in immunological cells (Nomellini et al., 2008). Additionally, the amount of catalase, superoxide dismutase, and glutathione peroxidase which are the enzymes responsible for free radical clearance in the cytosol are reduced in aged cells (Cannizzo et al., 2011). Similarly, the amount of manganese superoxide dismutase which is an antioxidant enzyme situated in the mitochondria, and defenses macrophages from apoptosis increased through oxidized low-density lipoprotein is also decreased in aging macrophages (Fujimoto et al., 2010) all of which contribute to the elevated level of cellular oxidative stress (Nomellini et al., 2008; Cannizzo et al., 2011).

## Vascular Aging

Vascular aging has been described in terms of changes in structure and function of the endothelium and smooth muscle cells and the communication routes between these two cell layers that form the vascular wall (Younger, 2004; Pase et al., 2012; El Assar et al., 2013). Impaired endothelial vasodilation is a virtual manifestation of arterial aging and a clinical indicator of vascular dysfunction, which may occur over a long period of time (Younger, 2004; Seals et al., 2006; El Assar et al., 2013). Moreover, impaired endothelial vasodilation is the first stage in changes in vascular outcomes and cardiovascular disease in elderly people (Seals et al., 2006; El Assar et al., 2013; van Buchem et al., 2014). With advancing age, there are changes that occur in the vasculature, including endothelial dysfunction, vascular remodeling, increased vascular stiffness and vascular inflammation, which contributes to hypertension (Grammas, 2011; Baierle et al., 2015; Harvey et al., 2015). In terms of hypertension, large and small arteries undergo mechanical structural, and functional changes which contribute to vascular complication and elevated cardiovascular risk (Savoia and Schiffrin, 2006; van Buchem et al., 2014). Moreover, the renin–angiotensin system exhibit a key role in the pathophysiology and development of hypertension and cardiovascular disease (Marchesi et al., 2008). Hypertension-enhanced vascular changes include low-grade inflammatory processes in which inflammation contributes to the pathophysiology of high blood pressure (Savoia and Schiffrin, 2006). One crucial hallmark of vascular aging is an increased arterial stiffness, resulting in the loss of arterial elasticity, compromising vascular adaptation to blood flow and pressure changes (El Assar et al., 2013; van Buchem et al., 2014; Pase et al., 2015). This increased arterial stiffness is often revealed *via* increases in the speed of propagation pressure/flow waves (Pase et al., 2010, 2012; El Assar et al., 2013). Arterial stiffness causes impaired endothelial vasodilation leading to endothelial dysfunction (Scuteri et al., 2008; Pase et al., 2010, 2015). Besides changes in the structure and function of the endothelium, endothelial dysfunction also has a crucial role in age-associated microvascular dysfunction (Rodríguez-Mañas et al., 2009). Small and large arteries are not isolated systems but are involved in crosstalk so that changes in the integrity and function of small arteries impact on the functionality of larger arteries, a condition that prevents small artery remodeling and organ damage (Laurent et al., 2009). This crosstalk between large artery changes and small cerebral arteries may be important in terms of cognitive aging (El Assar et al., 2013; van Buchem et al., 2014).

The functionality of the brain depends on a constant blood supply and disruptions in cerebral blood flow can lead to brain diseases and death (Moskowitz et al., 2010; van Buchem et al., 2014). Because of this, cerebrovascular control mechanisms function to ensure that the brain blood supply is sufficient for its energy requirements (Iadecola and Nedergaard, 2007). Increased neuronal activity is associated with an elevation in cerebral blood flow (functional hyperemia) which is thought to generate energy substrates and remove the toxic component that is derived from brain cellular activity (Paulson et al., 2010; Zlokovic, 2011). Cerebrovascular autoregulation manages cerebral blood flow to some degree ensuring some consistency in the range of blood pressure, and protecting the brain tissue from unwanted swings in perfusion pressure (van Beek et al., 2008). Specialized receptors on endothelial cells surface transduce mechanical (shear stress) and chemical stimuli, for instance endothelin, nitric oxide and prostanoids (Wolburg et al., 2009). Compelling evidence suggest that the health of brain is also dependent on the overall health of the cardiovascular system, and the existence of parenchymal and vascular inflammation which has often been offered as a link among atherosclerosis and AD (Grammas, 2011; Tzikas et al., 2014). To date, atherosclerosis and AD are known to share similar vascular risk factors for instance hypercholesterolemia, hypertension, as well as heart failure (Helzner et al., 2009; Roselli et al., 2009; Gorelick et al., 2016) and arterial stiffness (Triantafyllidi et al., 2009; Gorelick et al., 2016). The treatment of vascular risk factors has been shown to reduce the risk of developing Alzheimer’s disease (Babarskiene et al., 2002) and dementia (Jellinger, 2013) and to slow cognitive decline in AD patients (Deschaintre et al., 2009; Helzner et al., 2009). Compelling data also establishes a relationship between aortic stiffening and cognitive dysfunction (Pase et al., 2012; de la Torre, 2012) with greater cognitive impairment associated with poorer cerebral microcirculation and increased aortic stiffness (Triantafyllidi et al., 2009; Pase et al., 2010, 2012).

One of the key variations during vascular aging is the formation and development of inflame-aging (Franceschi, 2007). Inflamm-aging is not only independent of traditional cardiovascular disease risk factors but also accelerates arterial thickening and arterial stiffness independently from processes associated with cardiovascular disease (Scuteri et al., 2011). There is accumulating evidence for an increased systemic inflammatory mediators (inflamm-aging) for example IL-1β, TNF-α, members of the superfamily of IL-6, as well as, elevated amount of CRP in plasma of older adults while compared with young adults (Ferrucci et al., 2005). This increase in inflammatory markers is associated with age, and independent of other cardiovascular disease (Miles et al., 2008). The pleiotropic proinflammatory IL-6 has been importantly associated with age-dependent vascular disorders (Ungvari et al., 2004). Moreover, elevated plasma amount of IL-6 has been associated with larger disability and mortality in older people (Cesari et al., 2012). CRP levels are also correlated with elevated arterial stiffness in middle-aged and elderly (Mattace-Raso et al., 2004; Nakhai-Pour et al., 2007). However, expressions of MCP-1 and MMP are higher in the thickened arterial intima of vessels taken from autopsies of older people in comparison with those from young adult (El Assar et al., 2013). Vascular endothelial cells play a key role in the pathobiology of vascular inflammatory processes due to their potential interaction with elements related to systemic inflammation. They are potentially active participants during vasculitis, not only passive targets of injury (Younger, 2004). This is consistent with arterial changes that are associated with endothelial dysfunction (Scuteri et al., 2008; Pase et al., 2010, 2015). Several chronic vascular disorders are part of a progressive process, initiating and developing through local inflammation of large and medium sized arteries (Renna et al., 2013). It is scientifically relevant in this regard that pro-inflammatory signaling mechanism in the vascular wall has been well characterized and the risk of progressing age-associated neurodegenerative disease is related to increased circulatory inflammatory cytokines, such as IL-6 and TNF-α (Simen et al., 2011). Serum levels of homocysteine has an associated with atherosclerosis and can damage blood vessels. Crucially, AD disease data have demonstrated that higher levels of homocysteine independently and strongly predict the development of dementia (Fiolaki et al., 2014; Kim et al., 2018). VD is a heterogeneous group of brain disorders in which cognitive decline is attributable to cerebrovascular pathologies, and includes a large component of dementia prevalence (Gorelick et al., 2011; Iadecola, 2013). The level of some circulatory inflammatory proteins such as CRP and 1-antichymotrypsin have been observed to increase before the onset of VD (Engelhart et al., 2004). The level of CRP has also been shown to increase 25 years before the onset of VD (Schmidt et al., 2002). In the Conselice study of brain aging, with 4 years of follow-up, the combination of high levels of IL-6 and CRP led to a nearly three-fold increased risk of VD (Ravaglia et al., 2007). Yano et al. (2010) also reported that pentraxin 3, a circulatory inflammatory biomarker predicts cognitive decline in elderly hypertensive patients.

## What Is Vascular Inflammation (Vasculitis)?

Vasculitis is inflammation of the blood vessel wall, which causes different pathologies, depending on the type of impaired organ (Jennette and Falk, 1997; Hoffman and Calabrese, 2014). Vasculitis includes a heterogeneous class of disorders recognized *via* inflammation and necrosis of the blood vessel wall. Based on the Chapel Hill Consensus Conference, the primary systemic vasculitis could be characterized into three major groups influencing large-sized, medium- and small sized vessels, respectively (Jennette and Falk, 2007). Small sized vessel vasculitis is categorized under several names including Wegener’s granulomatosis, Churg–Strauss syndrome, Microscopic polyangiitis, Henoch–Schönlein purpura, Essential cryoglobulinemic Vasculitis, Cutaneous leukocytoclastic angiitis (Jennette and Falk, 1997). Medium-size arteries are involved in Kawasaki syndrome of childhood and in classic polyarteritis nodosa (Berlit, 2010). Large vessel vasculitis is giant-cell arteritis and Takayasu arteritis (Prieto-González et al., 2015). The cellular and molecular characterizations of vasculitis are complex and diverse, depending on the type of disorder and organ. Extensive reviews have been published on this topic (Younger, 2004; Rosenberg, 2009; Pantoni, 2010; Zuccoli et al., 2011; Hoffman and Calabrese, 2014; Pipitone et al., 2018).

## Vascular Inflammation and Cognitive Aging

The blood-brain barrier (BBB) is a dynamic and active barrier, selectively allowing the entrance of molecules (oxygen and nutrients) from blood into the brain and concomitantly protecting the brain form infections and blood toxins (Zlokovic, 2011). It consists of a monolayer of brain endothelial cells sealed with “tight junctions” which are proteinaceous transmembrane complexes made of members for instance; claudins, occluding and junctional molecule-1, and sub membrane molecules connecting to the actin-network (Jia et al., 2013). Endothelial cells of the BBB manage the neuronal environment through controlling the transport of numerous molecules from the blood to the brain parenchyma and vice versa. They also manage fluctuations *via* the synthesis of mediators able to impact on nerve cells function and vascular endothelial growth factor (Marchesi et al., 2008; Attems et al., 2011; Sanchez et al., 2013). Moreover, the BBB is permeable to pro-inflammatory mediators from systemic inflammation and permits leucocyte migration in to the brain (Lyman et al., 2014). Interestingly, vascular endothelial cells have a critical role in the pathobiology of vascular inflammation (Younger, 2004) and the variations in the metabolism of endothelial cells can cause neurodegenerative disorders. The neurovascular unit, which strongly influences neuronal cell activity of the brain (Bertini et al., 2013), also consist of astrocytes, and microglia which are the main neuroinflammatory principle cells (Seth and Koul, 2008; Ransohoff and Perry, 2009; Ransohoff and Cardona, 2010; Grammas, 2011), as well as pericytes and neurons (Grammas, 2011). Astrocytes connect to endothelial cells, providing support to those cells and as a result are able to regulate BBB maintenance they, act as mediators between BBB and neurons (Hawkins and Davis, 2005). Deregulation of the BBB, and neurovascular dysfunction has been observed to lead to neurodegeneration and cognitive decline (Iadecola, 2004; Zlokovic, 2011; Leung et al., 2013). Signaling cascade are correlated with angiogenesis and vascular activation and are up regulated in the micro vessels in brains of patients with AD leading to a complicated neuroinflammatory response, and neuronal synaptic disconnection, which can damage or kill the nerve cells (Zlokovic, 2008; Grammas, 2011; Sanchez et al., 2013), and consequently exhibit cognitive impairments (Ryan and Nolan, 2016). In AD human studies, there has been a significant elevation in inflammatory mediators in the cerebral microcirculation. Brain endothelial cells in AD express high level of inflammatory adhesion molecules, for instance cationic antimicrobial protein 37 kDa, MCP-1 and intercellular adhesion molecule-1 (ICAM-1; Frohman et al., 1991; Pereira et al., 1996; Grammas and Ovase, 2001). Moreover, AD brain micro-vessels express significantly higher amount and range of inflammatory mediators such as thrombin, nitric oxide, transforming growth factor-β, TNF-α, IL-1β, IL-6, IL-8 and MMPs (Dorheim et al., 1994; Grammas and Ovase, 2001, 2002; Thirumangalakudi et al., 2006). Crucially, inflammation has a key role in linking several vascular and neuronal damage to cardiovascular risk factors (Gorelick et al., 2011) such as arterial stiffness and hypertension (Gorelick et al., 2016). Inflammation is actively involved in cerebral vasculature, although the role of inflammation in vasculopathy is poorly understood (Klohs et al., 2014).

Unfortunately, most of the data available on vascular inflammation in relation to cognitive aging are based on animal models (Takeda et al., 2010; Yu et al., 2012; Won et al., 2013; Kaiser et al., 2014; Acharya et al., 2015) and available human studies mainly assess the role of systemic inflammatory biomarkers or symptomatic changes of cardiovascular risk factors in relation to cognitive aging. We describe here some of the studies that assist us in better understanding of the relationship between vascular inflammation and cognition. Several human studies have reported that platelet activity is significantly, correlated with dementia severity, supporting the role of vascular inflammation in the pathogenesis and progression of dementia (Laske et al., 2008, 2012; Stellos et al., 2010, 2014). Laske et al. (2008) suggested a correlation among soluble glycoprotein VI as markers of platelet activity with the pathogenesis of AD. Yano et al. (2010) reported that pentraxin 3, is a useful inflammatory biomarker for predicting cognitive decline in elderly hypertensive patients. Some human studies have also reported evidence that statins, cholesterol-lowering drugs have therapeutic application in AD (Jick et al., 2000; Wolozin et al., 2000) and patients in these studies exhibited a slower cognitive decline (Sparks et al., 2006). To investigate the relation among midlife hypertension and onset of AD later in life, Kruyer et al. (2015) chemically enhanced chronic hypertension in the transgenic Swedish-Dutch-Iowa mutation mouse model of AD. Hypertension increased cognitive impairments on the Barnes maze test and led to an increase in microvascular deposition of Aβ, vascular inflammation, BBB leakage, and pericyte loss. Moreover, hypertension enhanced hippocampal neurodegeneration at an early age in this mouse line, establishes this as a useful research model of AD with mixed amyloid and vascular pathologies (Kruyer et al., 2015). Grammas and colleagues reported that the vascular activation inhibitor, sunitinib, reduces Aβ as well as cerebrovascular expression of inflammatory proteins improved cognitive function in 3xTg-AD murine models and AD2576APP Swe (Grammas et al., 2014). Blocking angiotensin II signaling helps reduce neurodegeneration and to develop longevity in rodents (Benigni et al., 2010).

## Vascular Aging and Oxidative Stress

Inflammation and oxidative stress are underlying factors in the development of vascular aging and it is difficult to determine the effects of these two factors independently as several interplays co-exist between oxidative stress and inflammation and vice versa (Csiszar et al., 2008; Montezano and Touyz, 2014). Oxidative stress is crucially involved in several molecular and cellular interactions of vascular aging, which include: (1) elevated amount of pro-inflammatory responses in vascular cells; (2) vascular dysfunction among oxidative modification of structural and functional proteins which regulates vascular contraction/relaxation fibrosis and calcification; (3) variations in calcium homeostasis in vascular cells; (4) activation of redox-sensitive pro-inflammatory and profibrotic transcription factors; and (5) activation of molecular mechanisms causing senescence and autophagy in endothelial and vascular smooth muscle cells (Tatchum-Talom and Martin, 2004; Harvey et al., 2015).

## Brain Aging

To date, numerous functional and structural changes related to normal brain aging have been reported and indicated that brain mass reduces in the order of 2%–3% per decade after the age of 50, and in participants 80 years or older brain mass decreases 10% in comparison to young adults (Drachman, 2006). Voxel-based morphometry and magnetic resonance imaging has revealed that age particularly impacts on the volume of white and gray matter at parietal, prefrontal and temporal areas (Salat et al., 2004; Samanez-Larkin and Knutson, 2015; Von Bernhardi et al., 2015). Complex learning abilities, for instance, dual tasks (e.g., memorizing a word list while walking), show a progressive decline in the elderly (Salat et al., 2005; Von Bernhardi et al., 2015). However, cognitive decline during aging varies considerably, with some older people reporting normal cognitive abilities (Shock et al., 1984). Consistent with brain tissue and neuropsychological changes there are some brain changes that occur as a consequence of molecular and cellular changes in the body such as increases in the permeability of the BBB, enhancement in systemic inflammation, degeneration of neurons and other brain cells which could also lead to the production of ROS. It has been suggested that BBB permeability increases in aged animals (Blau et al., 2012; Enciu et al., 2013) facilitating infiltration *via* monocytes producing mitochondria-generated ROS (Zlokovic, 2008; Lyons et al., 2009). Microarray analysis of brain tissue provided from aged and young rodents have reported that the upregulated genes in the aged rodents are correlated with oxidative stress and inflammation (Lyons et al., 2009). In other research, enhancement in both major histocompatibility complexes (MHC)-II and glial fibrillary acidic protein (GFAP) were demonstrated in the brain of aged mice strengthening the hypothesis that activation of microglia and astrocytes are indicators of brain aging (Godbout et al., 2005) and all these changes could lead to chronic “neuroinflammation” (Lynch, 2009; Dong et al., 2014; Lyman et al., 2014).

## What Is Neuroinflammation?

Neuroinflammation is a complex cellular and molecular cascade in brain where immunological cells have a key role in its initiation and development (Glass et al., 2010; Skaper et al., 2014; Rizzo et al., 2014; Arulselvan et al., 2016). The BBB is one of several key players during neuroinflammation, where non-neuronal cells (e.g., microglia, asterocytes and pericytes) together with neurons form a functional unit, often referred to as a neurovascular unit to process a neuroinflammatory response (Iadecola, 2004; Hawkins and Davis, 2005; Zlokovic, 2005, 2008, 2011). BBB breakdown can occur under different conditions such as due to inflammatory responses, neurodegenerative processes, and vascular disorders. It can cause a neuroinflammatory response and generate neurotoxic products that cause a progressive synaptic disconnection, neuronal dysfunction and cell loss leading to a vicious circle in disorders such as AD, Parkinson’s disease (PD), multiple sclerosis (MS), and other disorders (Zlokovic, 2005, 2008, 2011). During the neuroinflammatory process, microglia and astrocytes are the main active immunological cells, which regulate both the enhancement as well as reduction of inflammatory production (Seth and Koul, 2008; Ransohoff and Perry, 2009; Ransohoff and Cardona, 2010). This condition is achievable *via* the synthesis of cytokines, up- or downregulation of several cell surface receptors such as pathogen recognition receptors, cytokine receptors, and several other receptors vital for antigen presentation (Ransohoff and Perry, 2009; Ransohoff and Cardona, 2010). Chronic neuroinflammation or upregulated neuroinflammation is commonly known *via* an enhancement in microglia activation and elevated level of inflammatory cytokines such as IL-1β, which are prevalent in almost all neurodegenerative diseases such as AD, MS, PD and amyotrophic lateral sclerosis (Lynch, 2009; Glass et al., 2010). Interestingly, these variations are similar, though less dramatic in the non-disease aging brain (Lynch, 2009). Supporting cells in the CNS (microglia and astrocytes) during neuroinflammation have a high turnover and exacerbate the process of brain cellular aging (Ma et al., 2013).

Research has also characterized the role of microglia during brain aging indicating a role of morphological, biological, physiological, anatomical, and molecular changes in aging microglia which also exhibit a comprehensive depiction of the senescent microglia phenotype (Norden and Godbout, 2013; Wong, 2013). This is due to the dynamic role of microglia cells in the CNS which have a crucial role in the development, plasticity and immune surveillance of the brain (Norden and Godbout, 2013). As such, we focus on the role of the microglia during neuroinflammation and brain aging. Microglia account for 10% of the total glial cell population in the brain. As a consequence of damage to the brain, microglia cells transform their morphology dramatically, migrating to the lesion sites and proliferate (Cerbai et al., 2012; Jurgens and Johnson, 2012; Norden and Godbout, 2013). Proliferated microglia cells phagocytose debris and dying cells and/or generate cytokines to support injured neurons and to keep the microenvironment homeostasis and are therefore useful for neuronal survival. Data has also shown a neurotoxic role of microglia whenever- they are activated in severe injury or neurodegenerative disorders (Jurgens and Johnson, 2012; Norden and Godbout, 2013). The two-way communication between neurons and microglia is vital for maintaining homeostasis during the physiological and chronic inflammatory response in the CNS. While microglia activation is crucial and beneficial in response to disorders and injury, unrestricted or prolonged activation could have harmful impacts on brain performance and behavior. To avoid inflammation-associated damage, microglia reactivity is modulated *via* neurons in the healthy brain (Jurgens and Johnson, 2012). Overall density and numbers of microglia have been shown to enhance notably with advancing age in several CNS compartments, which include the hippocampus (Wong, 2013) auditory and visual cortex (Tremblay et al., 2012) and the retina (Damani et al., 2011). These elevations might lead a low rate of basal microglia proliferation or in other respects i.e., to slow incremental recruitment of macrophages or monocytes from the periphery (Cerbai et al., 2012; Wong, 2013). From histopathology studies, some evidence has been provided that in the aged brain, microglia morphologies exhibit a perinuclear cytoplasm hypertrophy and retracted processes, almost similar to activated microglia (Miller and Streit, 2007; Wong, 2013). Aged microglia immunophenotypes also exhibit those of activated microglia, with enhanced expression of MHC II and complement receptor 3 (Frank et al., 2006; Ziv et al., 2006; Wong, 2013). In activated microglia, molecular markers are often observed to be up regulated, (such as ionized calcium-binding adaptor molecule1). Moreover, these markers are enhanced in aged microglia with no-evidence of injury or disease (Frank et al., 2006; Wong, 2013). During healthy aging, in human *in vivo* positron emission tomography (PET) using [11C]-PK11195 (TSPO radioligands) has shown an enhancement in ligand binding in several cortical and subcortical areas, demonstrating an elevated amount of basal microglia activation (Schuitemaker et al., 2012). Moreover, aged microglia express elevated amounts of effector molecules similar to activated microglia which is known to be the primary cell source of cytokines release such as TNF-α, IL-6, IL1-β (Lynch, 2009; Wong, 2013). Elevated expression of inflammatory cytokines (such as IL1-β, TNF-α, IL-6) are also elevated in aged microglia *in situ* (Wong, 2013), isolated *ex vivo* (Sierra et al., 2007; Njie et al., 2012) or when cultured *in vitro* (Ye and Johnson, 1999). Additionally, older brains show increased interaction among T cells and microglia (Lynch, 2009). Microglia senescence has an underlying role in the switching of microglia from neuroprotection in the young brain to neurotoxic in the aged (Norden and Godbout, 2013). Targeting some of these features of microglia senescence might constitute a feasible therapeutic strategy for some disorders and even cognitive aging itself (Jurgens and Johnson, 2012; Norden and Godbout, 2013; Wong, 2013). Overall, there is general agreement on the enhancement of basal state microglia activity in healthy aging, such that aged microglia may lead to chronic states of “para-inflammation” (Medzhitov, 2008) that is correlated with the enhanced vulnerability of the aged CNS to neurodegenerative disorders in which there is a crucial role of chronic neuroinflammation (Wong, 2013).

## Neuroinflammation and Cognitive Aging

Cognitive aging is characterized by a reduction in cognitive abilities in the elderly. Although the underlying mechanism involved in this process is not fully understood, neuroinflammation appears to be a significant contributor (Ownby, 2010). Several research studies have attempted to describe the relationship between neuroinflammation and cognition. To date, several animal studies (Hovens et al., 2014; Czerniawski et al., 2015; Elmore et al., 2015; Michels et al., 2015; Wei et al., 2015; Flannery et al., 2016; Laurent et al., 2017; Reis et al., 2017; Sanchez-Marin et al., 2017; Wang et al., 2018) have addressed this issue. For instance, a study by Sun et al. (2015) in male Sprague-Dawley rats showed that LPS could induce IL-17A, TNF-α, IL-6, iNOS, and COX-2 expression in the hippocampus when followed by IL-17A-neutralizing antibody treatment, and it significantly eliminated neuroinflammatory response through suppression of microglia activation and improved memory. Tian et al. (2015) also found that in a mice surgery model, partial hepatectomy enhanced the amount of IL-17A in the hippocampus and increased cognitive impairment, while vitamin D intervention reduced cognitive deficits by inhibiting Th17 cells and increasing T reg cell numbers. Tan et al. (2015) study also showed that transfusion of old red blood cells in Sprague-Dawley rats increased IL-6 in the hippocampus and enhanced ionized calcium-binding adapter molecule 1, in the cerebral cortex and hippocampus (neuroinflammatory response), and impaired memory and learning. Cognition and behavior were evaluated by Barnes maze and fear conditioning tests (Tan et al., 2015). A study by Hajiluian et al. (2017) with male Wistar rats model, showed that vitamin D reversed obesity-induced cognitive impairments *via* a reduction in nuclear factor kappa B (NFκB) amount (a key neuro-inflammatory factor) and an increase in brain derived neurotrophic factor concentration and modulation of the BBB permeability in the hippocampus. Cognitive function was examined by the Morris water maze test and the BBB permeability was evaluated by Evans blue dye in the hippocampus (Hajiluian et al., 2017).

The most widely applied transgenic Alzheimer animal models do not demonstrate the degree of inflammation in neurodegeneration and cognitive decline comparable to human disease. Therefore, a more suitable animal model, is required which closely mimics the resulting cognitive decline and memory loss in humans in order for us to be able to better understand the impact of neuroinflammation on neurodegenerative disorders (Millington et al., 2014). A few animal studies have specifically focused on the relationship between neuroinflammation and cognitive aging. For example, in the age-related Alzheimer mouse model, head injury can cause a chronic neuroinflammatory response, which initiates and causes cognitive impairment. A study by Webster et al. (2015) found that a single mild traumatic brain injury in the APP/PS1 knock-in mouse led to a delayed onset of neuroinflammatory response and a more persistent glia cells activation (microglia and astrocyte) in comparison to injured wild-type mice who consequently developed cognitive impairment (Webster et al., 2015). Fonken et al. (2016) demonstrated that high mobility group box 1 (HMGB1) mediates neuroinflammatory response priming in the aged brain of rats by blocking the HMGB1 action using a competitive antagonist Box-A. Aged-microglia became desensitized to an immunological challenge and therefore were prevented from an exaggerated neuroinflammatory response and sickness behavior following infection (Fonken et al., 2016). In the mice model of dementia 6-Shogaol (an active constituent of ginger) eliminated neuroinflammatory processes by inhibiting microgliosis and astrogliosis and consequently improved cognitive process (Moon et al., 2014).

**Figure 3:**
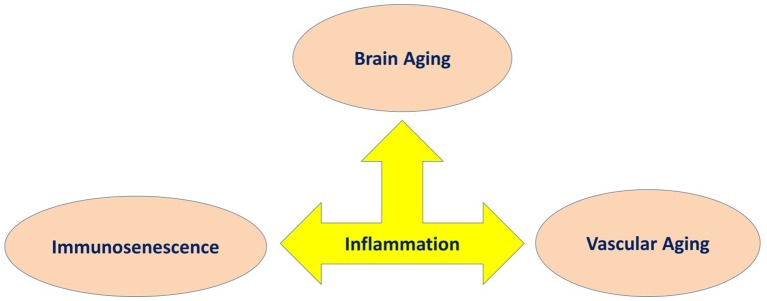
Inflammation as a common underlying mechanism in three main features of aging involved in cognitive decline.

PET has been frequently used in human studies to quantify microglia activity by labeling the translocator protein 18 kDa (TSPO), which becomes over expressed upon activation of microglia cells (Knezevic and Mizrahi, 2018). Studies that focus on neuroinflammation in MCI reported a similar association between cognitive impairment and TSPO binding (Okello et al., 2009; Yasuno et al., 2012; Schuitemaker et al., 2013). The only research with participants who had MCI reported a significant correlation among cognition and neuroinflammation was on the combined AD and MCI participants in the analysis (Kreisl et al., 2013a,b). There was a significant association among PET related inflammatory binding and impaired performance on the MMSE, logical memory immediate, clinical dementia rating, trail making part B tasks, and block design, with the strongest associations among [11C]-PBR28 (TSPO radioligands) binding in the inferior parietal lobule and performance on block design and clinical dementia rating score (Kreisl et al., 2013b). Three relevant studies have reported on relationships between neuropsychological assessments in AD patients and neuroinflammation (Kreisl et al., 2013b; Schuitemaker et al., 2013; Suridjan et al., 2015). In the study by Schuitemaker et al. (2013), there was no significant correlation between cognition and [11C]-PK11195 (TSPO radioligands) binding (Schuitemaker et al., 2013). However, two investigations with second-generation radioligands reported a significant association between performance on cognitive scales and TSPO binding. Kreisl et al. (2013b) reported a strong negative association between performance on the block design and [11C]-PBR28 (TSPO radioligands) binding in the inferior parietal lobule. Similarly, Suridjan et al. (2015) identified a negative correlation between performance on the visuo-spatial tasks in the repeatable battery for the assessment of neuropsychological status and [18F]-FEPPA (TSPO radioligands) binding in the parietal and prefrontal cortices. The former research additionally identified a negative association between [11C]-PBR28 binding and trail making part B task and performance on the logical memory immediate recall assesses executive function and memory, respectively. Three studies have reported that lower performance on the MMSE significantly correlated with higher levels of TSPO binding (Edison et al., 2008; Yokokura et al., 2011; Kreisl et al., 2013b). However, there are other studies that have demonstrated no association between these two variables (Yasuno et al., 2008; Schuitemaker et al., 2013; Varrone and Nordberg, 2015), and another study reported a positive association among MMSE score and the global cortical index (Hamelin et al., 2016). Based on the *in vivo* PET data, it can be concluded that chronic neuroinflammatory response plays a key role in the pathology of AD. Whether this chronic neuroinflammatory response is an initial factor or it happens consequentially during the development of disease is not known. The contradictory results across different studies using TSPO variables may be explained by methodological differences in the studies and how cognitive impairment was assessed. Future research should be conducted to include larger populations with longer observation, categorizing population *via* genetic, cognitive and behavioral phenotypes to study other factors, which could influence microglia activation (Figure 3).

## Discussion

Although, cognitive aging is a complex, multifactorial process, there are a number of key biological processes involved in this process. As such, we have proposed the involvement of three main processes that may explain cognitive aging, which includes immunosenescence, vascular aging and brain aging. Within this model, we described how they share common pathological processes such as inflame-aging, vascular inflammation and neuroinflammation, respectively (Figure 1). Capturing these three key processes in any single study is difficult for a number of reasons; however, their utility taken together should be better explored (Piazza et al., 2010; Cannizzo et al., 2011; Salvioli et al., 2013; Di Benedetto et al., 2017). We have also argued that immunosenescence is a critical key mechanism involved in cognitive aging (Figures 2, 3). Better understanding the development of immunosenescence in each individual should be further considered, and appears to be sensitive to both genetic and environmental factors (Weiskopf et al., 2009; Baylis et al., 2013; Di Benedetto et al., 2017). Immunosenescence affects how body regulate and manage different cellular and molecular interactions in almost all body organs (Markiewski and Lambris, 2007; Ownby, 2010; Fard et al., 2015; Arulselvan et al., 2016; Di Benedetto et al., 2017), particularly the brain during chronic neuroinflammatory processes. Neuroinflammation serves several fundamental roles in the brain structure and function, such as ion homeostasis, involvement in the regulation of metabolic function, the production of anti-oxidant species, synaptic levels of glutamate, modulation of neuroplasticity, maintenance of BBB, as well as protection from both endogenous and exogenous factors (Benarroch, 2005; Shih et al., 2006; Wang and Bordey, 2008; Broussard et al., 2012; Di Benedetto et al., 2017). We also propose that neuroinflammation is an underlying mechanism underpinning cognitive performance playing a vital role in learning and memory as we grow older (McAfoose and Baune, 2009; Ownby, 2010; Ryan and Nolan, 2016; Di Benedetto et al., 2017; Hajiluian et al., 2017). It is important to bear in mind that, there are normal or physiological levels of inflammation however, chronic events (Markiewski and Lambris, 2007; Fard et al., 2015; Sun et al., 2015; Tian et al., 2015; Arulselvan et al., 2016) may reshape a normal inflammatory response into an exaggerated response which could then be characterized in terms of inflamm-aging, vascular inflammation and chronic neuroinflammation. We therefore propose, that cognitive aging is a consequence of these three inflammatory processes which interact in a synergistic manner (Figures 2, 3). As such, treatments that ameliorate any or all aspects of exaggerated inflammatory responses may be beneficial in improving cognition or even ameliorating cognitive decline in the elderly. For instance it is commonly accepted that anti-inflammatory drugs are able to block important inflammatory pathways such as NF-κB, and mitogen-activated protein kinase (MAPK; Fard et al., 2015; Arulselvan et al., 2016) and these could be explored as treatments to ameliorate cognitive decline with age. Notably, whilst, nerve cells remain the most vital functional unit of the nervous system, immune cells acting directly or indirectly on brain function are more easily manipulated targets than nerve cells. Better understanding of the complex neuro-immune interactions will pave the way for the development of new therapies that target the immune system for the benefit of brain (Kipnis and Filiano, 2018), and cardiovascular function.

In general, immunosenescence is a pathological process, which manifests as a gradual decline in the functionality of our immune system over the lifespan at the molecular and cellular level and as such is hard to evaluate its initiation and development (Piazza et al., 2010; Cannizzo et al., 2011; Salvioli et al., 2013; Di Benedetto et al., 2017). Environmental factors such as life style and genetical factors directly influence immunosenescence in each induvial (Weiskopf et al., 2009; Baylis et al., 2013; Di Benedetto et al., 2017). Interestingly, a review by Kennedy et al. (2017) described the impact of exercise on improving cognitive functioning *via* several parallel pathways such as the modulation of inflammation and oxidative stress during brain aging. Another interesting report by Windham et al. (2014) on the associations between inflammation and cognitive function among two different population groups of African Americans and European Americans, demonstrated that population with high vascular risk, higher inflammation and poorer cognition were associated with markers of TNF-α activity and especially apparent in African Americans.

We have argued for the importance of three distinct, but inter-related inflammatory processes linked to cognitive aging such as inflame-aging, vascular inflammation and neuroinflammation (Figure 1). This model can be applied across different cognitive processes, however, not all older individuals show pathological inflammatory processes across these three domains at one time point. Chronic neuroinflammation may be the most directly relevant inflammatory domain in terms of cognitive aging, however inflamm-aging may be an important initiator of prolonged neuroinflammation. Finally, vascular inflammation may be more transient and specifically be related to cardiovascular disorders (Wilson et al., 2002; Zlokovic, 2005; Xie et al., 2009; Iadecola, 2010; Grammas, 2011; Broussard et al., 2012; Davenport et al., 2012; Kousik et al., 2012; Barrientos et al., 2015; Chi et al., 2016; Di Benedetto et al., 2017; Tarantini et al., 2017). In support of our model, there is evidence that some disorders show these relationships. For instance, in postoperative cognitive dysfunction (POCD) there is a significant relationship between systemic inflammatory mediators and neuroinflammation. A major operation can activate specific homeostatic reactions, which can generate an inflammatory response with the production of several inflammatory mediators (Hu et al., 2010; Terrando et al., 2011; Elwood et al., 2017). Damaged tissues activate monocytes, macrophages, fibroblasts and endothelial cells, and release various inflammatory products including oxidative radicals, complement split products and cytokines, such as IL-1, IL-6, TNF-α. These circulatory mediators can directly penetrate the BBB (Wilson et al., 2002). Cytokines bind to their receptors in the CNS, activate vascular endothelial cells and microglia cells, and subsequently, enhance neuroinflammatory response and a series of molecular reactions (Wilson et al., 2002; Xie et al., 2009). This neuroinflammatory response can lead to cognitive decline, which could impact on the production and activity of neurotransmitters, decrease neural plasticity, and increase neurotoxicity (Hu et al., 2010). Interestingly, a review by Cunningham and Hennessy (2015) noted that a growing body of clinical, preclinical and epidemiological evidence shows that chronic co-morbidities and systemic inflammatory contributes to the progression of dementia. VD is known to be correlated with cerebrovascular pathologies (Gorelick et al., 2011; Iadecola, 2013). Recent data, has revealed a role of cerebrovascular disorders, not only as initial cause of cognitive decline, but also as an adjuvant to the expression of dementia in AD and other neurodegenerative pathologies (Iadecola, 2013). Vascular risk factors impair the structure and function of cerebral blood vessels and the neurovascular unit, and these pathological changes are then mediated *via* vascular oxidative stress and inflammation (Iadecola, 2010). In the review by Sharp et al. (2011), hypertension was shown to be an important risk factor in cerebrovascular disease, cognitive impairment and AD as well as, for VD. Moreover, VD is associated with microinfarcts of cerebral blood vessels preventing oxygen supply to neurons which is caused to some extent because inflammation of the arterial wall affects the accumulation of thrombotic factor (Libby et al., 2009). Accumulating evidence also suggests that neuroinflammation is a hallmark of AD and several studies have shown the existence of biomarkers of neuroinflammation, in brain tissue of AD patients (e.g., inflammatory cytokines, chemokines, and activated microglia (Eikelenboom et al., 2010; Lee et al., 2010; Sudduth et al., 2013; Klohs et al., 2014; Morales et al., 2014; Hoeijmakers et al., 2017). Epidemiological studies have also shown that long-term consumption of non-steroidal anti-inflammatory drugs inhibits the progression of AD and delay its onset, suggesting that there is a significant relation among AD pathogenesis and neuroinflammation (Holmes et al., 2009; Lee et al., 2010) (Figure 1).

## Inflammation and Cognitive Impairment in Dementia

Cognitive aging is a generic concept, with more than 100 diseases leading to dementia (Vijayan and Reddy, 2016), while we discussed the three specific inflammatory domains including neuroinflammation supported by three types of dementia such as POCD, VD, and AD. Our proposed model can be applied to other types of pathological cognitive aging, as well. It also seems plausible to hypothesize high commonality in the three inflammatory domains outlined across cognitive aging (particularly chronic neuroinflammation which is common feature of all dementias). As we discussed earlier, the specific inflammatory hallmark in each dementia varies depending on the type of dementia. We now focus on some of these issues in more detail.

The involvement of cellular senescence and inflammatory process *via* complex molecular and cellular processes occur in neurons and gilal cell in PD and MS patients have been reviewed previously by Kritsilis et al. (2018). Neuroinflammation is one of the mechanisms involved in PD-cognitive impairment. Elevation in cortical microglia activation has been reported in 11 PD-dementia patients, using biological parametric mapping analysis (Fan et al., 2015). Cerebrospinal fluid level of cytokines was observed to be correlated with PD-cognitive impairment (Lindqvist et al., 2013). Petrou et al. (2016) found correlations among diabetes, gray matter loss and PD-cognitive impairment using magnetic resonance imaging in 36 patients, possibly due to neuroinflammation caused by mitochondrial dysfunction (Aviles-Olmos et al., 2013; Petrou et al., 2016). In a human study by Hall et al. (2018), inflammatory biomarkers such as CRP and serum amyloid A in cerebrospinal fluid were higher in PD-dementia elderly individuals compared to elderly individuals without PD. In addition, inflammation was associated with more motor symptoms and cognitive decline (Hall et al., 2018).

MS is an inflammatory neurodegenerative disease of the CNS mainly effecting young adults. In this condition, infiltrating myelin-reactive lymphocytes (mainly T-cells but also B-cells) attack axon antigens and myelin sheaths on oligodendrocytes and neurons in the CNS. These insults cause a neuroinflammatory cascade, formation of large demyelinating plaques in the white matter and gliosis, and synaptopathy, leading to an impairment of the neuronal signaling and, later on to neurodegeneration (Dendrou et al., 2015; Mandolesi et al., 2015; Musella et al., 2018). Clinical characteristics include motor impairments, sensory and visual disturbances, pain, fatigue, mood disturbances and cognitive deficits (Dendrou et al., 2015). MS patients frequently suffer from cognitive impairment and it estimated between 40% and 65% of MS patients tend to progress cognitive impairment gradually over time. Cognitive impairment is present in MS patient with progressive clinical onset compered to patients in relapsing remitting phase (Huijbregts et al., 2004; Ruet et al., 2013; Planche et al., 2016; Matias-Guiu et al., 2017), although some heterogeneous results have been reported as well (Rao, 1990; Potagas et al., 2008). In MS patients during immunosenescence and possibly due to inflame-aging, additional inflammatory responses cause chronic neuroinflammatory responses which therefore significantly accelerate CNS aging (Dendrou et al., 2015; Musella et al., 2018). Several studies have also suggested that an exacerbation of common neuropathological aspects of aging brain and MS such as neuroinflammatory processes, synaptic dysfunction, cellular loss (synaptopathy) and synaptic plasticity impairment (Di Filippo et al., 2008; Weiss et al., 2014; Mandolesi et al., 2015; Stampanoni Bassi et al., 2017; Musella et al., 2018), may explain the effect of aging on MS disability. A recent study by Surendranathan et al. (2018) reported that both neuroinflammatory responses with microglia activation and peripheral inflammatory changes are observed in dementia with Lewy bodies. A review by Swardfager et al. (2018) recently indicated that an increased risk of dementia in severe psychological disorders such as major depressive disorder and bipolar disorder is associated with inflammation, oxidative stress and variations in metabolic pathways. Moreover, those pathways are involved in the premature development of metabolic and vascular comorbidities (Swardfager et al., 2018).

Patients who survive a stroke are also at high risk of recurrent microvascular changes. The mechanisms that underpin this process is not fully understood. A recent data showed that stroke-enhanced atherosclerosis is induced by brain-released biochemical which lead to vascular inflammation and plaque formation (Rust et al., 2018). Inflammation in stroke appear to have both beneficial and detrimental effects (Jin et al., 2010). Chronic inflammatory response may trigger neurotoxic pathways leading to progressive degeneration. Damaged neurons also may exacerbate neuroinflammation-mediated disorders by producing chemokines and activation of microglia (Zhang and Yang, 2014). Numerous recent longitudinal studies have examined the correlation among inflammatory biomarkers and post-stroke dementia but a relationship is not yet established. Erythrocyte sedimentation rate, CRP, IL-6, IL-12 were also suggested as predictors of post-stroke cognitive impairment (Rothenburg et al., 2010; Narasimhalu et al., 2015). Kliper et al. (2013) showed strong relationship between cognitive performance and erythrocyte sedimentation rate between stroke survivors, where higher erythrocyte sedimentation rate levels were correlated with poorer performance in cognitive tests, particularly memory scores. Preliminary data has also demonstrated that cortical amyloid deposition and post-stroke white matter neuroinflammation contribute to post-stroke dementia (Arboix et al., 2014). Interestingly, Malojcic et al. (2017) argued that cerebral hypoperfusion is linked to cognitive decline either as an aggravating factor or risk factor. Hypoperfusion as a consequence of macroangiopathy, microangiopathy, or cardiac dysfunction may also promote or accelerate neuroinflammation, BBB disruption and neurodegeneration.

Obesity is correlated with low grade systemic inflammation, peripheral insulin resistance and high oxidative stress (Chunchai et al., 2016; Saiyasit et al., 2018). Moreover, obesity can cause neurodegeneration and cognitive impairment *via* induction of hippocampal inflammation, hippocampal mitochondrial dysfunction, hippocampal insulin resistance, and hippocampal oxidative stress (Chunchai et al., 2016; Stranahan et al., 2016; Saiyasit et al., 2018). These changes also are associated with a decline in hippocampal synaptic plasticity and the number of dendritic spines (Sa-Nguanmoo et al., 2018; Saiyasit et al., 2018). Moreover, obesity induced through high-fat-diet consumption, leads to an elevation in amyloid plaque formation and neuronal cell death (Kothari et al., 2017; Pintana et al., 2017). Growing evidence demonstrates a close relationship among type 2 diabetes Mellitus and neurodegenerative diseases such as AD. They share several pathological characteristics comprising inflammation, oxidative stress, brain vasculopathy, impaired insulin sensitivity, tau hyper-phosphorylation, and amyloid accumulation (Tumminia et al., 2018). Riederer et al. (2017) posited that peripheral inflammatory biomarkers in diabetes mellitus can pass BBB and initiate neuroinflammation and microglia activity, thus contributing to the pathophysiology of AD and VD.

There is also data that supports the notion that a reduction in vascular functions and brain metabolism occur decades before the onset of cognitive impairments and these reductions are highly associated with chronic neuroinflammation that develop over time. Crucially, recent findings suggest that the gut microbiota (GMB) play a vital role in modulating immune reactions in the brain through the brain-gut axis (Hoffman et al., 2017). Some recent data has indicated that a specific subset of the GMB can stimulate neuroinflammation in rodents (Erny et al., 2015; Palm et al., 2015; Petra et al., 2015) and influence brain function and behavior in rodents and humans (Li et al., 2009; Bercik et al., 2011; Diaz Heijtz et al., 2011). A study by Hoffman et al. (2017) found that inflammation influences neurovascular function, brain metabolism, gut microbiome, memory and anxiety in aging mice. Interestingly, a study by Cattaneo et al. (2017) in elderly humans indicated that an elevation of a proinflammatory GMB taxon, Escherichia/Shigella, and a decrease in the abundance of an anti-inflammatory taxon, E. rectale, are correlated with systemic inflammatory biomarkers in patients with cognitive impairment and brain amyloidosis. This finding is in line with the hypothesis that GMB composition may drive peripheral inflammation, leading chronic neuroinflammatory responses, brain amyloidosis and probably, neurodegeneration and cognitive decline in AD (Cattaneo et al., 2017).

## Pharmacological Approaches

Current treatment paradigms for neurodegenerative disorders such as dementia are limited by their significant side-effects and poor long-term efficacy, creating an essential need to develop preventative therapies that target common pre-symptomatic risk factors such as inflammation and oxidative stress. There are a large number of pharmacological agents applied to improve cognitive decline by managing inflammation and oxidative stress; however, in this section we briefly summarize the key published studies on this topic (Herman et al., 2018).

Phytochemicals are able to interfere with the NF-κB pathway and manage inflammation. They suppress the ubiquitination or phosphorylation of signaling molecules, and therefore, supress the degradation of IκB. The translocation of NF-κB to the nucleus and subsequent transcription of pro-inflammatory cytokines are suppressed *via* the actions of phytochemicals. In addition, natural bio-compounds that prevent the interaction of NF-κB can block NF-κB’s transcriptional activity by suppressing its binding to target DNA. Several polyphenols such as curcumin, pterostilbene, resveratrol, macranthoin G, punicalagin, salidroside, 4-O-methylhonokiol, genistein, lycopene, gallic acid and obovatol have been reviewed as potent NF-κB inhibitors for AD treatment by Seo et al. (2018). Several alkaloids including galantamine, tetrandrine, glaucocalyxin B, oridonin, berberine, anatabine have also shown anti-inflammatory properties in AD models *in vitro* as well as *in vivo*. Moreover, vitamins (such as vitamin-D, alfa-Tocopherol quinine, Retinoic acid), artemisinin, tanshinone IIA, geniposide, dihydroasparagusic acid, xanthoceraside, 1,8-cineole, L-theranine, and paeoniflorin were posited as promising NF-κB inhibitors (Seo et al., 2018).

An interesting review by Skvarc et al. (2018) has summarized several novel therapies with common anti-inflammatory properties in different stages of preclinical and clinical levels as therapeutic targets to deal with POCD such as Parecoxib/COX-II inhibitors, Statins, Pregabalin, Dexmedetomidine, Lidocaine, Ketamine, Minocycline, and N-Acetylcysteine.

Vinpocetine [14-ethoxycarbonyl-(3a,16a-ethyl)-14,15-eburnamine] is a synthetic derivative of vinca alkaloid vincamine which is an alkaloid extracted from the periwinkle plant, *Vinca minor* (Gulyás et al., 2002a,b). Currently, vinpocetine is available in many countries as a dietary supplement to improve cognition and memory. Moreover, it has been clinically used in several countries for treatment of cerebrovascular disorders such as stroke and dementia. Zhang et al. (2017) has argued that vinpocetine is a potent anti-inflammatory agent based on different *in vitro* cell culture models. By directly suppressing IKK activity, and enhancing the stability of IκB, that leads to binding of IκB with NF-κB and subsequent suppression of NF-κB dependent inflammatory molecule expression (Jeon et al., 2010; Zhang et al., 2017). Vinpocetine can penetrate the BBB and enter the brain (Gulyás et al., 2002a,b). The anti-inflammatory effects of vinpocetine has also been demonstrated in several animal models *in vivo*. In a rat cerebral ischemia-reperfusion injury model, NF-κB and TNF-α level were found to be correlated with changes in brain edema and infarct volume. Vinpocetine suppressed NF-κB and TNF-α expression and reduced the inflammatory response after cerebral ischemia-reperfusion (Wang et al., 2014). More importantly, the anti-inflammatory effect of vinpocetine was recently reported in a study involving 60 patients with anterior cerebral circulation occlusion and onset of stroke (Zhang et al., 2018). Participants treated with vinpocetine not only had a better recovery of psychoneurological function and improved clinical outcomes, but also had reduced NF-κB signaling activation and pro-inflammatory biomarker expression (Zhang et al., 2017).

A recent review by Masoumi et al. (2018) has also summarized evidence for the hypothesis that Apelin neuropeptide is an effective and comprehensive therapeutic agent to improve cognitive function in AD, and unlike current therapies, can influence a broad range of molecular mechanisms involved in AD pathogenesis. Apelin reduces the accumulation of Aβ and decreases phosphorylation and accumulation of tau protein. Apelin also prevents neurodegeneration by suppressing production of inflammatory mediators, especially TNF-α, IL-6 and IL-1β, which plays an important role during neuroinflammatory process and the pathogenesis of AD. Apelin can modulate N-methyl D-aspartate receptors and therefore decrease excitotoxicity and death of neurons and prevents neuronal apoptosis. In addition, it exhibited high antioxidant properties preventing free radicals and ROS productions. Apelin also enhances synaptic plasticity of the neurons, and improves cognitive function and memory by increasing factors such as endothelial nitric oxide, angiotensin converting enzyme-2 and Glucagon-like peptide-1 (Masoumi et al., 2018). Also see other reviews on this topic (Rodriguez-Grande et al., 2017; Solas et al., 2017; Meeusen and Decroix, 2018; Zhong et al., 2018; Fish et al., 2019).

## Limitations and Future Research

Studies examining the involvement of the three main inflammatory types posited by us in this review (inflame-aging, vascular inflammation, and neuroinflammation) in cognitive processes are mainly hampered by the lack of direct studies involving humans. While animal studies help us understand the cellular and molecular mechanism of cognition, human clinical studies are still needed given the differences in complexity and range of cognition that can be studies between animals and humans. Another major limitation of the current literature is that there are only a few sensitive biomarkers for inflammation in humans with the available markers specifically reflecting molecular and cellular processes rather than brain related inflammation. Another challenge in studying the biological processes that underpin cognitive aging are that processes such immunosenescence and chronic inflammation have a progressive and time dependent nature, which require observation over a long time. This is obviously, expensive from a clinical perspective. Cognitive aging itself is a complex and multifactorial disorder, which requires the investigation of diverse molecular and cellular processes.

## Conclusion

In developed countries, there is a significant increase in the proportion of older citizens. Increasing number of older citizens pose a number of critical issues for our societies including increased cognitive aging, increased numbers of patients with AD and a loss of independence and reliance on social security. Therefore, a better understanding of the role of processes such as inflammation in cognitive and brain aging is important. Immunosenescence is a pathological phenomenon and a central concept that brings together our understanding of age-associated chronic disorders, functional decline and aging across the lifetime. A better understanding of the cellular and molecular mechanisms underlying brain aging, as well as their potential interactions, provides a growing list of factors that can be targeted for specific interventions aimed to prevent or delay cognitive decline associated with aging. Future studies on the role of inflammation during cognitive aging are necessary and would benefit from directly addressing the three types of inflammation described in this review article.

## Author Contributions

Both authors were involved in all aspects of the conceptualization and writing of the manuscript.

## Conflict of Interest Statement

The authors declare that the research was conducted in the absence of any commercial or financial relationships that could be construed as a potential conflict of interest.
